# Hybrid methods combining atmospheric reanalysis data and a parametric typhoon model to hindcast storm surges in Tokyo Bay

**DOI:** 10.1038/s41598-019-48728-7

**Published:** 2019-08-21

**Authors:** Fei Liu, Jun Sasaki

**Affiliations:** Department of Socio-Cultural Environmental Studies, Graduate School of Frontier Sciences, The University of Tokyo, 5-1-5 Kashiwanoha, Kashiwa, Chiba 277-8563 Japan

**Keywords:** Natural hazards, Physical oceanography, Hydrology

## Abstract

Reanalysis data and a parametric typhoon model formula are often used to prepare wind and pressure fields for storm surge hindcasting. However, their optimum selection and usage have not been well established. To enhance the accuracy of wind and pressure fields, two hybrid methods were proposed by applying a parametric typhoon model of Mitsuta–Fujii’s formula, which is determined from the typhoon center to a certain radius of *R*_b_, and then switching to the reanalysis data of ERA-Interim in the outer region through the interpolated transition bandwidth of *W*_b_. In hybrid model I, *R*_b_ and *W*_b_ were fixed because the time-varying radius of the maximum wind speed was determined by the typhoon formula. In hybrid model II, these parameters were determined to minimize the mean difference between the reanalysis data and the fields obtained by typhoon formula in the transition band at each time step of the typhoon track. The hindcasting of eight significant typhoon events approaching Tokyo Bay was performed. This validated the proposed methods in comparison with the observed storm surge anomalies. Both models performed satisfactorily. Hybrid model II was found to be superior in terms of the balance of accuracy and preparation cost.

## Introduction

**Figure 1:**
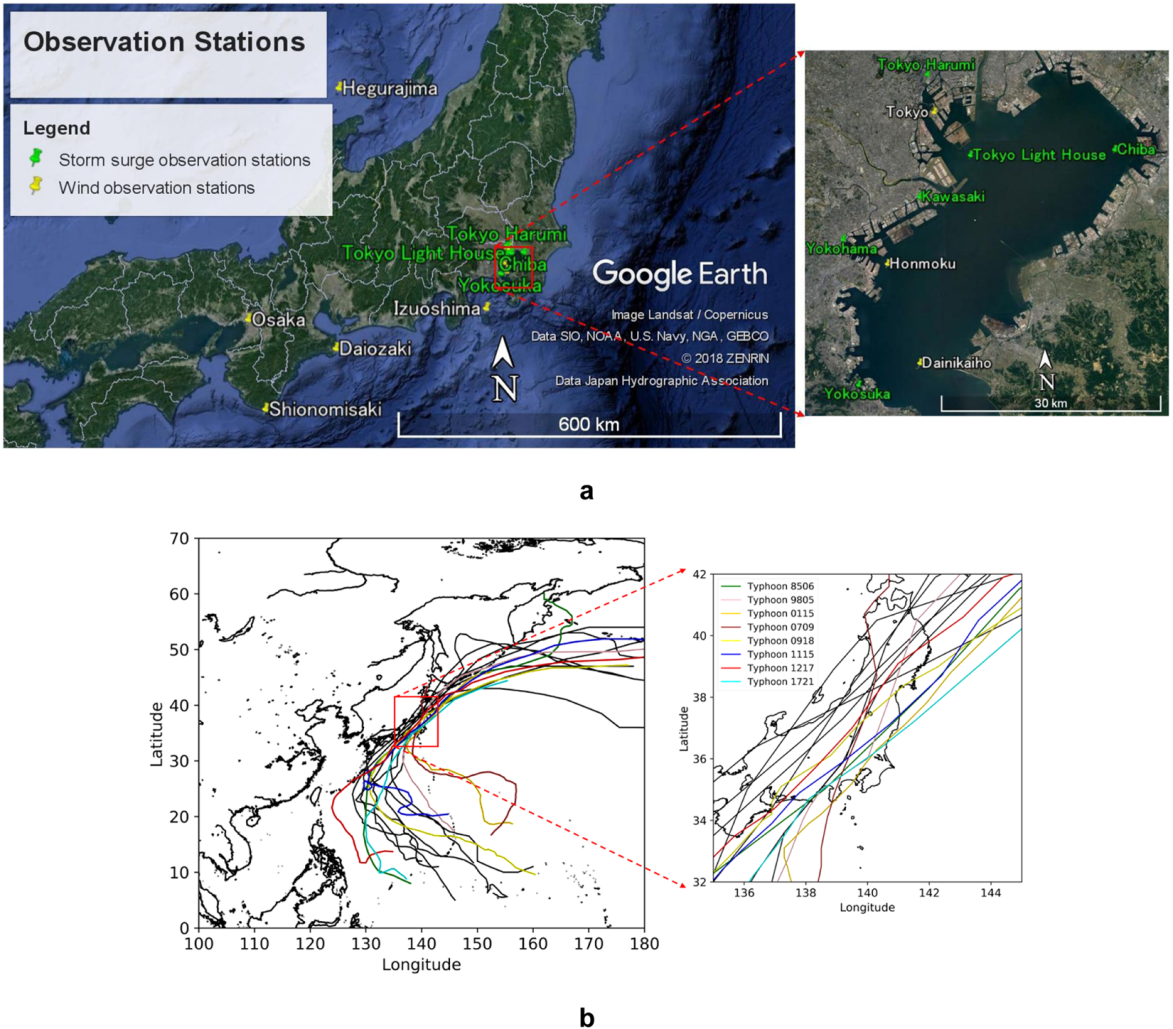
(**a**) Map of Tokyo Bay and the western region of Japan with meteorological and tide observation stations. (**b**) Historical typhoon tracks causing significant storm surge anomalies in Tokyo Bay from 1951 to 2017.

Tokyo Bay is a semi-enclosed coastal sea in the Greater Tokyo area, one of the largest city areas in the world, with a population of over 37 million (Fig. 1). Tokyo Bay area has been exposed to coastal hazards including storm surges^1–3^ and tsunamis^4–7^. In particular, storm surges are considered to be the most significant coastal disasters at the bay head where Tokyo Port and large commercial and residential areas are situated. According to a report by Chiba Prefecture Government^8^, the highest possible storm surge anomaly is projected to be 5.7 m at the bay head. The typhoon in the 6th year of the Taisho era (1917) caused one of the historical storm surges in Tokyo Bay, resulting in overflow of the coastal defense system and leading to extensive damage to properties and a loss of more than 1,300 lives^1^.

The accuracy of wind and atmospheric pressure data is important to storm surge hindcasting. A wide variety of parametric typhoon models have been developed to reproduce realistic atmospheric pressure and wind fields during typhoons or tropical cyclones, such as the Takahashi formula^9^, Fujita formula^10^, Jelesnianski formula^11^, Myers formula^12^, Mitsuta–Fujii formula^13^ (M–F), and Holland formula^14^. In these models, the maximum wind radius (*r*_max_) that controls the maximum wind speed is a key parameter in determining the wind field, specifically the peak wind speed which could be estimated using several empirical formulae^15–17^. In contrast, the long-term reanalysis wind data obtained from data assimilation models has been widely used for storm surge hindcasting owing to its global availability and convenience. The data provided by the National Centers for Environmental Prediction (NCEP)^18,19^, European Center for Medium-Range Weather Forecasts (ECMWF)^20,21^, and Japanese Reanalysis (JRA)^22,23^ are the most commonly used reanalysis datasets. However, previous studies^24–28^ have shown that the wind speeds obtained near the typhoon center from the reanalysis data cannot accurately reproduce the observed wind fields. Thus, modifications are needed. Chao *et al*.^26^ proposed a framework to blend data from NCEP’s operational Global Forecast System (GFS) with that from a higher-resolution hurricane prediction model and successfully demonstrated an appropriately blended wind field for the typhoon-generated wind-wave prediction. However, the proposed method seems complex and impractical. Shao *et al*.^27^ presented two critical values as applicable ranges for ECMWF reanalysis wind data and Holland formula application for typhoon events that occurred in the South China Sea and East China Sea. With these two critical values, a weighting coefficient was given to combine two sets of wind data; however, the weighting coefficient was not clearly explained. Pan *et al*.^28^ proposed a similar superposition method and applied it to Typhoon Fanapi and Typhoon Meranti in 2010 by analyzing the cross-calibrated multi-platform (CCMP) reanalysis wind data. However, their method was not validated in storm surge hindcasting.

Thus, the objective of this study is to propose a practical method for creating accurate wind and atmospheric pressure fields by blending a reanalysis dataset and a parametric typhoon formula. The widely used ERA-Interim wind and atmospheric pressure reanalysis dataset (ERA-I) and M–F model were employed. Two hybrid methods for creating the fields were proposed; their performances were validated using the observed meteorological data provided by the Japan Meteorological Agency^29^ (JMA). Using the created atmospheric boundary data, hindcasting of two historical storm surges in Tokyo Bay was performed using the unstructured-grid Finite Volume Community Ocean Model^30^ (FVCOM 4.1), which has been applied to storm surge computations in several studies^31–37^.

The remainder of this paper is organized as follows. In Section 2, two hybrid methods are described and validated by comparison with the observed wind and atmospheric pressure fields, and the storm surge hindcasting is performed for the cases of Typhoon 8506 (the 6th typhoon in 1985) and Typhoon 1115 (the 15th typhoon in 2011) in Tokyo Bay, demonstrating the performance of the proposed hybrid methods. Section 3 presents the discussion and conclusions. Section 4 presents materials and methods.

## Results

By introducing the radius of *R*_b_ and the transition bandwidth of *W*_b_, a generalized hybrid method is proposed where the M–F model is applied in the 0 ≤ r ≤ *R*_b_ region and switching to ERA-I in the *r* ≥ *R*_b_ + *W*_b_ region (outer region of the transition band) while interpolating the two models in the transition band of *R*_b_ < *r* < *R*_b_ + *W*_b_. In Section 2.2, this generalized idea (hybrid model II) is introduced with a method to determine the *R*_b_ and *W*_b_ parameter values. Before that, as a special case, hybrid model I is introduced in Section 2.1 by applying the M–F model in the region between the typhoon center (*r* = 0) and the applicable boundary, *r* = *r*_max_, and switching to ERA-I in the region where *r* ≥ 2*r*_max_. ERA-I and M–F model were smoothly interpolated in the transition band of *r*_max_ < *r* < 2*r*_max_.

### Hybrid model I

The direct method of determining the wind and atmospheric pressure field is given by:

$${F}_{{\rm{B}}}={F}_{{\rm{M}}-{\rm{F}}}\,(0 < r\le {r}_{{\rm{\max }}})$$

1 $${F}_{{\rm{B}}}=\frac{2{r}_{{\rm{\max }}}-r}{{r}_{{\rm{\max }}}}{F}_{{\rm{M}}-{\rm{F}}}+\frac{r-{r}_{{\rm{\max }}}}{{r}_{{\rm{\max }}}}{F}_{{\rm{ERA}}}\,({r}_{{\rm{\max }}} < r\le 2{r}_{{\rm{\max }}})$$

$${F}_{{\rm{B}}}={F}_{{\rm{ERA}}}\,(r > 2{r}_{{\rm{\max }}})$$

where *F*_B_ is the resultant wind velocity component in x or y direction, or the atmospheric pressure, *F*_M−F_ and *F*_ERA_ are the wind velocity or atmospheric pressure from M–F model and ERA-I, respectively, *r* is the distance between the target location and typhoon center, and *r*_max_ is given by equation (6). Compared with the method of Shao *et al*.^27^, the proposed hybrid model can be easily used, and the weighting coefficient is explicitly explained.

Comparing the wind velocity distributions between the ERA-I and hybrid model I (see Supplementary Fig. S1), ERA-I and the M–F model dominated outside and inside the typhoon region, respectively, and the transition between these two models was continuous.

Comparisons between the computed (ERA-I, M–F model, and hybrid model I) and observed (JODC) wind speeds are shown in Supplementary Fig. S2. Their coefficients of determination are presented in Table 1. It can be seen that wind speeds computed by hybrid model I were more consistent with those of the observed (JODC) values during Typhoon 8506 and Typhoon 1115 than those computed by the M–F model or ERA-I.

**Table 1:** Comparison of *R*^2^ value (coefficient of determination) for ERA-I, M–F model, hybrid model I, and hybrid model II for Typhoon 8506 and Typhoon 1115.

Station	M–F	ERA-I	Hybrid model I	Hybrid model II
**R** ^**2**^ **value (Typhoon 8506)**				
Tokyo	0.584	0.498	0.704	0.682
Honmoku	0.464	0.334	0.469	0.479
Hegurajima	0.345	0.591	0.605	0.592
Shionomisaki	0.145	0.386	0.405	0.416
**R** ^**2**^ **value (Typhoon 1115)**				
Tokyo	0.405	0.270	0.547	0.609
Honmoku	0.464	0.457	0.529	0.512
Dainikaiho	0.492	0.370	0.506	0.539
Daiozaki	0.514	0.263	0.520	0.559

### Hybrid model II

**Figure 2:**
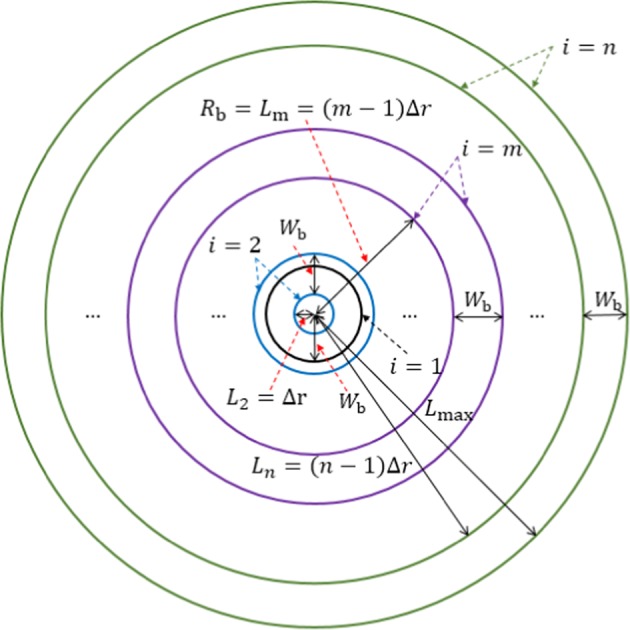
Searching band areas in hybrid model II (the first parameter is the increase in the searching distance Δ*r*, the second parameter is the maximum searching radius *L*_max_, the third parameter is the bandwidth *W*_b_, which is the equal for all searching bands, and the fourth parameter is the distance from the typhoon center to the inner side of the transitional annulus band area *L*_*i*_ (*i* = 1, 2, …, *n*). *R*_b_ equals *L*_*m*_ if the *m*^th^ annulus is the band area within which the difference of ERA-I data and the M–F model is minimum. The searching begins from the first circle (black color) *i* = 1 with radius *W*_b_, then the blue color annulus *i* = 2 with internal radius *L*_2_ = Δ*r*, etc. The investigation stops when *L*_*n*_ + *W*_b_ = 1000 km. It is clear that in the proposed searching method, part of the neighboring band area is overlaps because the searching band area gradually expands with Δ*r*).

The second hybrid model is based on the analysis of the difference between ERA-I and the M–F model. As shown in Section 2.1, the accuracy of the M–F model is higher than that of ERA-I around the typhoon center while the accuracy of ERA-I is higher than that of the M–F model away from the typhoon center. Thus, an optimum switching method between the M–F model in the central part and ERA-I in the outer typhoon region was obtained by interpolating the two data in the transition band at each time step for the typhoon track. At each time step, both the distance *r* = *R*_b_ from the typhoon center to the inner side of the transition band and the bandwidth *W*_b_ were investigated from *r* = 0 until *r* ≤ *L*_max_ (*L*_max_ is the maximum searching length) to minimize the mean value of the differences between ERA-I and M–F model across the computational grids within the transition band. For simplicity, a variable of the searching increment, Δ*r*, was introduced where *R*_b_ = (*m* − 1)Δ*r* (*m* is an integer number and 1 ≤ *m* ≤ *n*) and the maximum integer number of *n* was determined to satisfy (*n* − 1)Δ*r* = *L*_max_ − *W*_b_ (see Fig. 2). The formula for hybrid model II is given by:

$${F}_{{\rm{B}}}={F}_{{\rm{M}}-{\rm{F}}}\,(0 < r\le {R}_{{\rm{b}}})$$

2 $${F}_{{\rm{B}}}=\frac{{R}_{{\rm{b}}}+{W}_{{\rm{b}}}-r}{{W}_{{\rm{b}}}}{F}_{{\rm{M}}-{\rm{F}}}+\frac{r-{R}_{{\rm{b}}}}{{W}_{{\rm{b}}}}{F}_{{\rm{ERA}}}\,({R}_{{\rm{b}}} < r\le {R}_{{\rm{b}}}+{W}_{{\rm{b}}})$$

$${F}_{{\rm{B}}}={F}_{{\rm{ERA}}}\,(r > {R}_{{\rm{b}}}+{W}_{{\rm{b}}})$$

The increment value of Δ*r* was set as 5 km and the maximum searching length of *L*_max_ was set as 1000 km, considering the balance between the accuracy and computational cost. Supplementary Fig. S3 shows the time series of the optimum *R*_b_ to *r*_max_ ratio for ten cases of *W*_b_ to obtain the optimum bandwidth. The ratios were always 0 when the distances from the typhoon center to the computational nodes were greater than 1000 km, e.g., in the period until 18:00 on June 28^th^. For all ten cases, the ratios were generally less than 2, which is consistent with the applicable radius, *r*, range for the M–F model (0 ≤ *r* ≅ *r*_max_) as suggested by hybrid model I.

Comparisons between the computed winds (ERA-I, M–F model, and hybrid model II) and the JODC measured data during Typhoon 8506 and Typhoon 1115 are shown in Supplementary Fig. S4. Their coefficients of determination are summarized in Table 1, including the results of hybrid model I. According to Table 1, in some cases, such as Station Tokyo for Typhoon 8506 and Station Honmoku for Typhoon 1115, hybrid model I performed better than hybrid model II. In other cases, such as Station Honmoku for Typhoon 8506 and Station Tokyo for Typhoon 1115, hybrid model II was more accurate than hybrid model I. Thus, results show the accuracies of these two methods varied with cases and stations.

To further evaluate the performance of hybrid models I and II, eight selected typhoon cases were analyzed, and their corresponding root mean square errors (RMSE) were calculated by:

3 $$RMSE=\sqrt{\frac{1}{N}{\sum }_{i=1}^{N}{({F}_{{\rm{hb}}-{\rm{I}},i}-{F}_{{\rm{JODC}},i})}^{2}}$$

where *F*_hb−I,*i*_ is the wind velocity value or atmospheric pressure at the *i*^th^ time step during a certain typhoon computed by hybrid model I, *F*_JODC,*i*_, is the corresponding measured wind speed or atmospheric pressure, and *N* is the total number of typhoon time steps. Supplementary Table S1 presents comparisons of the RMSE values among ERA-I and hybrid models I and II with different bandwidths. Compared with the original ERA-I RMSE values, both hybrid models were found to improve accuracy. However, the results from other cases showed that hybrid model II performed better with varying optimal bandwidths, compared with hybrid model I. Hybrid model I was considered a special case of hybrid model II where the bandwidth and band distance from the typhoon center were determined based on ERA-I and M–F model. Although the computational cost for hybrid model II was slightly higher, its performance was better and thus, would be a better choice for enhancing the accuracy of storm surge computation.

The hybrid model II concept was then applied to the atmospheric pressure fields, which is also vital in enhancing the storm surge computation accuracy. Comparisons of the time series of atmospheric pressures at Station Tokyo and Station Yokohama for Typhoon 8506 and Typhoon 1115 between ERA-I, Myers formula, hybrid model II, and observed data are shown in Supplementary Fig. S5. The blended atmospheric pressures obtained by hybrid model II were more consistent with those of the measured values. Thus, hybrid model II is confirmed to be superior.

### Application to hindcast storm surge

**Figure 3:**
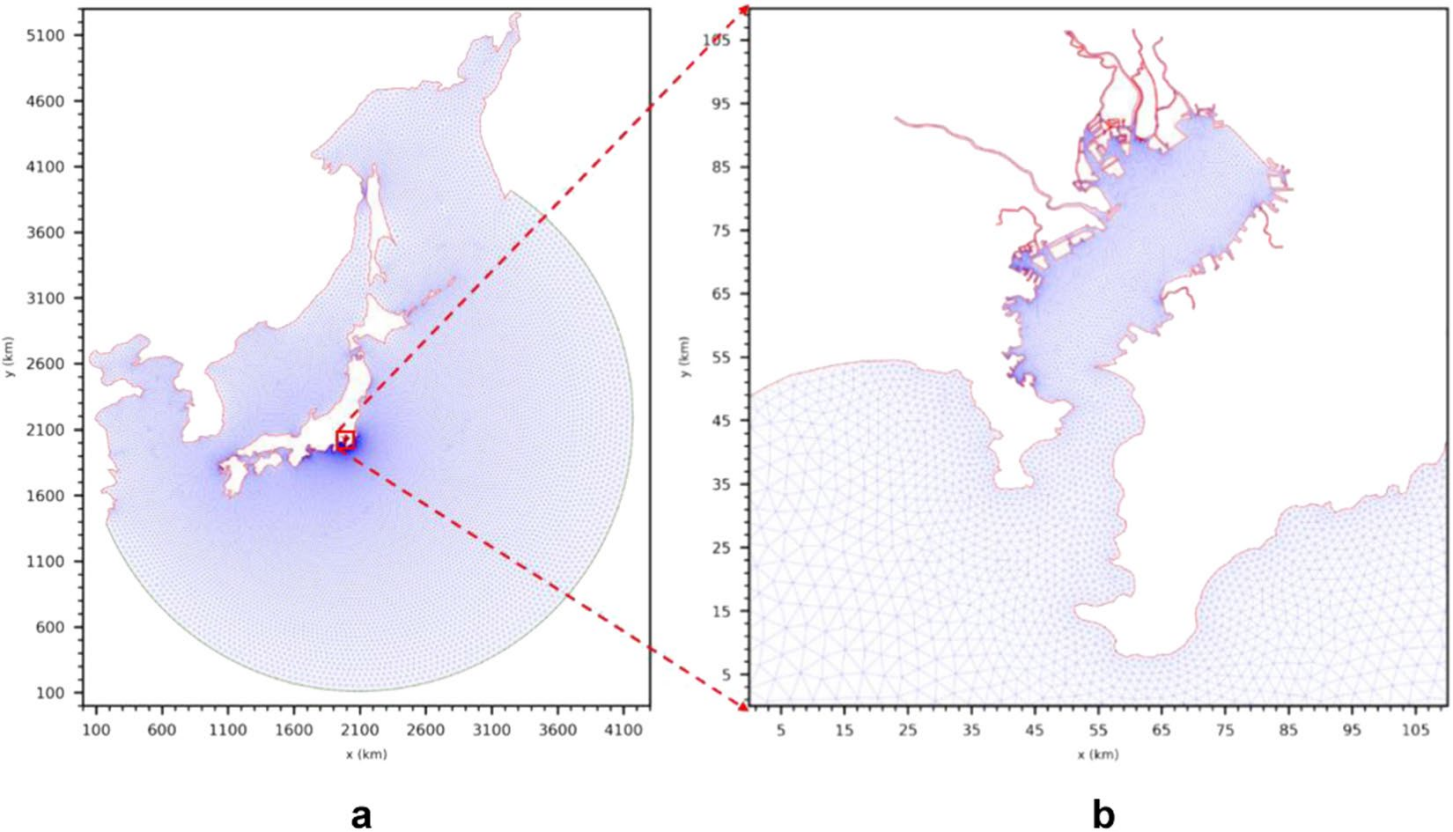
Triangular mesh for the study domain. (**a**) Entire computational domain. (**b**) Tokyo Bay area.

To validate the performance of the proposed hybrid models for wind and atmospheric pressure fields, a hindcasting of storm surges in Tokyo Bay was performed using the finite volume community ocean model (FVCOM) forced by the wind and pressure data of ERA-I, M–F model with Myers formula, and the two hybrid models. To further consider the effect of possible water volume exchange between the ocean from a far place and the inner side of the bay, and also to reduce the boundary influence, a wide area was selected as the computational domain (20°N–60°N, 120°E–160°E) as shown in Fig. 3. A total of 34,255 computational nodes and 61,905 triangular elements were used.

Supplementary Table S2 summarizes the information on the storm surge observation stations^38,39^, and Fig. 1a shows the station locations in Tokyo Bay. To compare the performance of the hybrid wind data, the same set of parameters and boundary conditions were adopted except for the wind and atmospheric pressure data. The computation started with the still water condition with a mean sea level and zero velocities across the domain. For the wind data, ERA-I, M–F model, hybrid model I, and hybrid model II were used. For the atmospheric pressure data, Myers formula, ERA-I, and hybrid model II were applied.

Supplementary Fig. S6 shows the comparisons of the storm surge anomalies between the hindcasting results and observed data at six stations in Tokyo Bay for Typhoon 8506. Before the storm surge peaked at 5:00 on July 1^st^, 1985, the observed sea level anomalies at six stations were generally higher than the mean sea level (=0). ERA-I reproduced the observed tendency better than the M–F model with Myers formula. This indicates the significant influence of the atmospheric pressure fields on the reproducibility of sea level anomalies before the typhoon approach. However, ERA-I data substantially underpredicted the storm surge peak at all six stations, which implies the insufficient spatial resolution of ERA-I data to reproduce the surge peak. Comparing all the model results, hybrid model II (for both wind and atmospheric pressure fields) performed better in reproducing the storm surge anomalies during the typhoon approach, the surge peak, and the following oscillations induced by bay-scale resonance.

Supplementary Fig. S7 shows comparisons of the storm surge anomalies between the computed values using six models and the observed values for Typhoon 1115 at four stations in Tokyo Bay. M–F model could not reproduce the storm surge anomalies well before the peak, and the peak anomalies in Station Tokyo Harumi, Station Chiba, and Station Yokosuka were significantly lower than the observations. Similarly, ERA-I could not reproduce the peak anomalies at four stations. Among the six storm surge models, only hybrid model II (for both wind and atmospheric pressure fields) exhibited better results in reproducing storm surge anomalies.

To further evaluate the accuracy among the six models, the RMSE (defined by equation (3)) between the computed and observed values are presented in Table 2 for Typhoon 8506 and Typhoon 1115. The following are the periods of comparisons: from 6:00 on June 29^th^ to 9:00 on July 2^nd^, 1985, for Typhoon 8506, and 3:00 on September 20^th^ to 23:00 on September 23^rd^, 2011, for Typhoon 1115. According to Table 2, it can be concluded that the wind and atmospheric pressure fields obtained by hybrid model II should be used to hindcast storm surges.

**Table 2:** Statistics for storm surge simulation results of different models for Typhoon 8506.

Stations	Typhoon 8506 RMSE (m)					ERA-I
	M–F model I	M–F model II	Hybrid model I	Hybrid model II	Hybrid model II (*U* and *P*)	
Tokyo Harumi	0.172	0.155	0.140	0.133	0.098	0.214
Tokyo Light House	0.223	0.126	0.113	0.099	0.084	0.176
Chiba	0.243	0.213	0.191	0.192	0.121	0.242
Yokohama	0.153	0.137	0.122	0.122	0.064	0.147
Yokosuka	0.136	0.106	0.092	0.091	0.053	0.093
Kawasaki	0.200	0.137	0.134	0.138	0.083	0.156
**Stations**	**Typhoon 1115 RMSE (m)**					**ERA-I**
	**M–F model I**	**M–F model II**	**Hybrid model I**	**Hybrid model II**	**Hybrid model II (** ***U*** **and** ***P*** **)**	
Tokyo Harumi	0.290	0.213	0.189	0.209	0.113	0.266
Tokyo Light House	0.256	0.159	0.158	0.144	0.121	0.221
Chiba	0.242	0.227	0.204	0.226	0.117	0.258
Yokosuka	0.195	0.137	0.120	0.117	0.071	0.150

## Discussion and Conclusions

Parametric typhoon models and reanalysis datasets are widely used for storm surge simulation. However, only a few studies seek their optimum combination to enhance the accuracy of storm surge computation. In this study, two hybrid models were proposed for wind fields, where the M–F model was applied between the typhoon center (*r* = 0) and a certain radius (*r* = *R*_b_), and switched to ERA-I when the radius is greater than *R*_b_ + *W*_b_ through the linearly interpolated transition band with the width of *W*_b_. For hybrid model I, both *R*_b_ and *W*_b_ were fixed as the time-varying radius of *r*_max_ was determined by the M–F model at a distance from the typhoon center where the maximum wind speed occurred. Conversely, in hybrid model II, the optimum combination of *R*_b_ and *W*_b_ was determined to minimize the mean difference between the M–F model and ERA-I within the transition band. Thus, hybrid model II was considered to be the generalization of hybrid model I. The wind fields of the eight historical typhoon cases approaching Tokyo Bay were compared between ERA-I, the M–F model, hybrid model I, and hybrid model II. The accuracy of the typhoon wind fields calculated by ERA-I, the M–F model, hybrid model I, and hybrid model II were verified by comparison with the observed wind field provided by JODC. Results showed that while both hybrid models performed better than ERA-I and the M–F model, the accuracy of hybrid model II was higher than that of hybrid model I. The atmospheric pressure fields were also modified using hybrid model II.

Using FVCOM, hindcasting of storm surges in Tokyo Bay was performed by forcing six combinations of modeled wind and atmospheric pressure fields. The results showed that the modification of both atmospheric pressure and wind fields significantly improved the accuracy of the storm surge anomalies. Both proposed hybrid models performed better than the computations using only ERA-I or the M–F model. Hybrid model II can be easily tuned for each storm surge cases using only ERA-I and the M–F model data, which significantly improved the accuracy of storm surge hindcasting.

Forecasting the storm surge a short time period ahead of the typhoon’s arrival was out of the scope of this paper. However, the proposed method may be applied to the storm surge prediction under future climate conditions. The future atmospheric fields could be prepared using a general circulation model (GCM) with future climate scenarios, e.g., scenarios discussed in Intergovernmental Panel on Climate Change (IPCC) reports. Then, these atmospheric fields can be used in place of ERA-I reanalysis data for hybrid model I and II combining with parametric typhoon model.

## Materials and Methods

### Typhoon cases and observation stations

The locations of the meteorological observation stations in Tokyo Bay and its surrounding areas are shown in Fig. 1a. The stations recorded the tide levels, including the storm surge anomaly, and wind speed in a one-hour time interval. The detailed information, including the station names, longitudes, and latitudes, is summarized in Supplementary Tables S2 and S3.

Among historical typhoons and storm surges that occurred in Tokyo Bay between 1951 and 2017, 15 typhoon cases with large storm surge anomalies (greater than 0.8 m) were screened out and eight typhoons (see Fig. 1b) were selected as summarized in Supplementary Table S4.

### Wind data analysis

Several reanalysis datasets are for wind and pressure fields, including the ERA-I, NCEP-DOE reanalysis II, and parametric typhoon models. A preliminary investigation was performed on the data consistency by a comparison with observed data from eight typhoon cases obtained in Section 4.1. ERA-I and the M–F model were used because both of them were more consistent with the measured data for the eight typhoon cases in Tokyo Bay during the preliminary study. Moreover, this method is applicable to any combination of reanalysis datasets and typhoon formulas.

#### ERA-Interim reanalysis data

ERA-Interim (ERA-I) is a global atmospheric reanalysis production provided by the European Center for Medium-Range Weather Forecasts (ECMWF), which started in 1979 and has since been continuously updated. In this study, ERA-I data of 6-hour interval wind speeds at a 10-m height from the mean sea level and the sea surface level atmospheric pressure were used. One-hour interval time series datasets for ERA-I were also created by interpolation. To obtain the most accurate ERA-I at the target stations, a 0.125° grid in the longitude and latitude was used.

**Figure 4:**
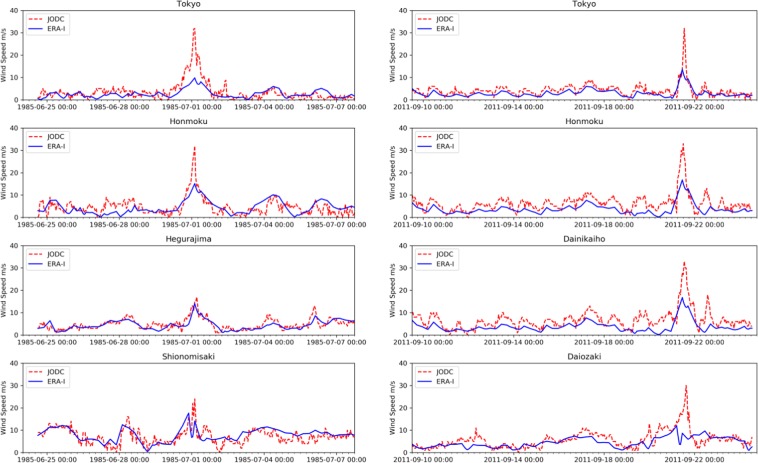
Time series comparison of ERA-I and JODC measured wind data during Typhoon 8506 (left) and Typhoon 1115 (right).

Figure 4 shows a comparison of ERA-I and the measured wind data provided by Japan Oceanographic Data Center^40^ (JODC) at four stations during Typhoon 8506 and Typhoon 1115. When the center of Typhoon 8506 was far from Station Tokyo (1985/06/24/06:00-1985/07/01/00:00 and 1985/07/01/10:00-1985/07/03/06:00), Station Honmoku (1985/06/24/06:00-1985/06/30/18:00 and 1985/07/01/10:00-1985/07/07/00:00), and Station Shionomisaki (1985/06/24/06:00-1985/06/29/12:00 and 1985/07/02/12:00-1985/07/07/00:00), ERA-I was consistent with that of the measured JODC data. When the center of Typhoon 8506 approached Station Tokyo (1985/07/01/00:00-1985/07/01/10:00), Station Honmoku (1985/06/30/18:00-1985/07/ 01/10:00), and Station Shionomisaki (1985/07/01/00:00-1985/07/01/10:00), ERA-I was lower than that of the measured JODC data. For Typhoon 1115 and the other six typhoon cases, similar analyses were carried out.

With the typhoon center far from the stations, the wind speeds were fairly consistent with the observed data with the corresponding approaching and departing distances between the typhoon center and Station Tokyo, Station Honmoku, and Station Shionomisaki ranging from 3.94 *r*_max_ to 1.84 *r*_max_ (1985/7/1/0:00-1985/7/1/10:00), 3.09 *r*_max_ to 2.23 *r*_max_ (1985/6/30/18:00-1985/7/1/10:00), and 2.15 *r*_max_ to 2.44 *r*_max_ (1985/6/29/12:00-1985/7/1/12:00), respectively. With the aforementioned distances, large discrepancies between the observed data and ERA-I were noted.

After the ERA-I analysis for the eight typhoon events and the JODC observation data at eight stations, the applicable ranges for ERA-I are summarized in Table 3. The critical value, *D*_1_, is defined as the applicable distance limit from the typhoon center. It was proposed to identify the applicable range of ERA-I at a target location during the typhoon approach. ERA-I is applicable at a location when the distance from the typhoon center to the location is greater than *D*_1_. A similar explanation can be applied to *D*_2_, which is defined for the typhoon departure condition. The distances *D*_1_ and *D*_2_ consist of the applicable boundaries for ERA-I.

**Table 3:** Critical values of *D*_1_–*D*_4_ at eight stations under eight selected Typhoons.

Typhoon	Station	*D*_1_/*r*_max_	*D*_2_/*r*_max_	*D*_3_/*r*_max_	*D*_4_/*r*_max_
8506	Tokyo	3.940	1.840	1.550	0.880
8506	Honmoku	3.090	2.230	0.810	0.970
8506	Hegurajima	3.610	1.560	1.730	1.410
8506	Shionomisaki	2.150	2.440	1.090	1.153
9805	Izuoshima	1.580	1.760	0.890	1.000
9805	Daiozaki	1.470	1.810	1.060	0.800
0115	Honmoku	2.080	2.050	1.200	0.928
0709	Tokyo	2.036	2.079	1.070	1.110
0918	Tokyo	2.300	2.460	0.850	1.360
0918	Osaka	1.950	2.860	1.480	1.530
1115	Tokyo	3.000	2.160	0.980	0.608
1115	Honmoku	2.790	2.400	1.068	1.150
1115	Daiozaki	1.930	2.600	0.89	0.730
1115	Dainikaiho	2.770	1.830	1.050	0.600
1217	Tokyo	2.530	1.997	0.970	0.870
1217	Honmoku	1.530	1.800	0.910	0.950
1217	Dainikaiho	2.430	2.110	0.910	1.000
1217	Izuoshima	2.152	2.076	0.750	0.760
1217	Daiozaki	1.770	1.790	1.680	1.100
1721	Tokyo	2.140	2.720	0.590	0.540

The first step in determining *D*_1_ and *D*_2_ values is the plotting of the time-series ERA-I data and JODC observed data at each station during the selected typhoon events. Instances with large discrepancies are then noted. The distance from the typhoon center to the station at the noted instance is finally calculated as the *D*_1_ value for this typhoon. The distance of *D*_2_ is similarly determined under the departing condition. As shown in Table 3, different stations experienced the whole processes of the eight selected typhoon events. Statistics at the eight stations under different typhoon events resulted in different *D*_1_ values. It can be seen that *D*_1_ and *D*_2_ values are approximately 2 *r*_max_. Hence, to simplify the following analysis, *D*_1_ and *D*_2_ are set as 2 *r*_max_.

#### Parametric typhoon model description

The Myers formula based on the exponential distribution of the atmospheric pressure field is given by:

4 $$P(r)={P}_{{\rm{c}}}+({P}_{0}-{P}_{{\rm{c}}}){e}^{-\frac{{r}_{{\rm{\max }}}}{r}}$$

where *P*(*r*) is the pressure at a radial distance *r* from the typhoon center, *P*_c_ (hPa) is the typhoon central pressure, *P*_0_ (=1013.25 hPa) is the ambient or environmental pressure, *r* is the distance from the computational mesh node to the typhoon center, and *r*_max_ (km) is the maximum wind speed radius.

After reviewing similar studies^17,41^, the M–F model was selected to compute the wind field as presented in equation (5) and the estimated pressure by equation (4) was applied to the M–F model.

$${U}_{{\rm{w}}}(r)=\,{U}_{{\rm{w}}1}(r)+{U}_{{\rm{w}}2}(r),$$

5 $${U}_{{\rm{w}}1}(r)={C}_{1}(-\frac{fr}{2}+\sqrt{{(\frac{fr}{2})}^{2}+\frac{r}{{\rho }_{{\rm{a}}}}\frac{\partial P}{\partial r}}),\,$$

$${U}_{{\rm{w}}2}(r)={C}_{2}\frac{{U}_{{\rm{w}}1}(r)}{{U}_{{\rm{w}}1}({r}_{{\rm{\max }}})}{V}_{{\rm{T}}}$$

*U*_w_ is the total wind vector, *U*_w1_ is the moving component, *U*_w2_ is the wind vector induced by the rotating component, *P*(*r*) is the pressure field calculated by Myers formula, *C*_1_ and *C*_2_ are dimensionless coefficients ranging from 0.6 to 0.75, *f* is the Coriolis parameter, *r* is the distance from the typhoon center, *ρ*_a_ is the atmospheric density, and *V*_T_ is the typhoon forward speed obtained from the best track data^29^ provided by the JMA including the typhoon center location and central pressure. The time-varying radius of the maximum wind speed, *r*_max_, was determined as a function of central pressure, *P*_c_, following the empirical formula^17^:

$${r}_{{\rm{\max }}}=0.769{P}_{{\rm{c}}}-650.55,\,{\rm{when}}\,880\,{\rm{hPa}} < {P}_{{\rm{c}}}\le 950\,{\rm{hPa}}$$

6 $${r}_{{\rm{\max }}}=1.633{P}_{{\rm{c}}}-1471.35,\,{\rm{when}}\,{P}_{{\rm{c}}} > 950\,hPa$$

Applying this model to the eight typhoon cases, the estimated wind speeds were compared with the observed data for Typhoon 8506 and Typhoon 1115 cases as shown in Supplementary Fig. S2. In general, for large wind speeds, the estimated values were consistent with that of the measured data. Following Section 4.2.1, the applicable boundary distances of *D*_3_ and *D*_4_ were determined for the M–F model from the typhoon center to the target location when the typhoon is respectively approaching and departing, as shown in Table 3 (*D*_3_ and *D*_4_ are approximately equal to *r*_max_). Thus, M–F model is applicable in the area between the typhoon center (*r* = 0) and the boundary (*r* = *r*_max_).

## Supplementary information

Supplementary Information
