# Supplementary material for: Hybrid methods combining atmospheric reanalysis data and a parametric typhoon model to hindcast storm surges in Tokyo Bay
Source: Sci Rep. 2019 Aug 21;9:12222. doi: 10.1038/s41598-019-48728-7 (PMC6704263; doi:10.1038/s41598-019-48728-7)
Supplement: Supplementary file 1 — Supplementary Information [file 41598_2019_48728_MOESM1_ESM.pdf]

Supplementary Information

Hybrid methods combining atmospheric reanalysis data and a parametric typhoon model to hindcast storm surges in Tokyo Bay

Fei LIU[1, *], Jun SASAKI[2]

[1] Department of Socio-Cultural Environment Studies, Graduate School of Frontier Sciences, the University of Tokyo, 5-1-5 Kashiwanoha, Kashiwa, Chiba 277-8563, Japan. fei2019.liu@gmail.com

[2] Department of Socio-Cultural Environment Studies, Graduate School of Frontier Sciences, the University of Tokyo, 5-1-5 Kashiwanoha, Kashiwa, Chiba 277-8563, Japan. jsasaki@k.u-tokyo.ac.jp

Corresponding author: Fei LIU

Mailing address: Department of Socio-Cultural Environment Studies, Graduate School of Frontier Sciences, the University of Tokyo, 5-1-5 Kashiwanoha, Kashiwa, Chiba 277-8563, Japan

Tel: +81-80-30258886

E-mail: fei2019.liu@gmail.com; 5564443124@edu.k.u-tokyo.ac.jp

Supplementary Table S1 | Comparison of RMSE values between hybrid model I and hybrid model II with different bandwidths

Typhoon	Station	Root Mean Square Error (RMSE) (m/s)											
		5 km	10 km	20 km	25 km	50 km	100 km	200 km	250 km	500 km	1000 Km	Hybrid model I	ERA-I
8506	Tokyo	3.096	3.088	3.083	3.080	2.917	2.871	2.694	2.548	2.511	2.807	2.397	3.392
8506	Honmoku	3.040	3.002	2.948	2.941	2.945	2.981	2.954	2.944	2.963	2.967	2.964	3.181
8506	Hegurajima	1.673	1.673	1.673	1.673	1.673	1.673	1.663	1.654	1.673	1.779	1.640	1.653
8506	Shionomisaki	3.099	3.099	3.081	3.037	2.972	2.972	2.948	2.942	2.919	2.960	2.943	2.981
9805	Izuoshima	2.451	2.451	2.451	2.451	2.353	2.192	2.252	2.326	2.500	2.688	2.503	3.109
9805	Daiozaki	2.648	2.648	2.648	2.648	2.609	2.595	2.593	2.575	2.566	2.947	2.484	2.675
0115	Honmoku	2.837	2.774	2.381	2.382	2.406	2.418	2.322	2.239	2.593	2.731	3.055	2.731
0709	Tokyo	1.711	1.711	1.711	1.725	1.702	1.784	1.791	1.799	1.869	2.094	2.051	1.986
0918	Tokyo	1.990	2.000	1.997	2.000	1.940	1.708	1.667	1.667	1.770	1.977	1.682	1.977
0918	Osaka	2.076	2.076	2.076	2.076	1.988	1.895	1.876	1.863	2.014	2.175	1.845	2.175
1115	Tokyo	2.645	2.645	2.580	2.498	2.402	2.321	2.282	2.279	2.249	2.294	2.308	2.789
1115	Honmoku	3.754	3.763	3.768	3.769	3.778	3.710	3.688	3.684	3.696	3.685	3.619	3.879
1115	Daiozaki	3.438	3.438	3.438	3.438	3.323	3.091	3.227	3.233	3.158	3.551	3.338	3.511
1115	Dainikaiho	3.827	3.827	3.827	3.827	3.801	3.669	3.665	3.666	3.667	3.718	3.758	4.602
1217	Tokyo	1.728	1.728	1.717	1.716	1.718	1.719	1.711	1.691	1.718	1.783	1.745	1.761
1217	Honmoku	3.247	3.247	3.247	3.247	3.247	3.213	3.218	3.200	3.218	3.352	3.206	3.258
1217	Dainikaiho	3.414	3.414	3.414	3.414	3.414	3.417	3.427	3.404	3.405	3.494	3.359	4.355
1217	Izuoshima	3.038	3.038	3.038	3.038	3.038	3.036	3.051	3.061	3.057	3.149	3.092	3.237
1217	Daiozaki	3.525	3.525	3.525	3.525	3.525	3.378	3.370	3.370	3.366	3.514	3.063	3.420
1721	Tokyo	2.174	2.153	2.150	2.151	2.154	1.875	1.852	1.850	1.939	2.031	2.203	1.877

Supplementary Table S2 | Storm surge observation stations

Station name	Longitude (°)	Latitude (°)	Typhoon number	Source
Tokyo Harumi	139.766667	35.666667	8506, 1115	JMA*
Tokyo Light House	139.828056	35.566111	8506, 1115	Bureau of Port and Harbor TMG*
Chiba	140.045556	35.568056	8506, 1115	Bureau of Port and Harbor TMG*
Yokohama	139.633333	35.466667	8506	JMA*
Yokosuka	139.651389	35.288056	8506, 1115	JCG*
Kawasaki	139.75	35.516667	8506	JMA*

*JMA: Japan Meteorological Agency; TMG: Tokyo Metropolitan Government; JCG: Japan Coast Guard

Supplementary Table S3 | Meteorological observation stations

Observation stations	Longitude (°)	Latitude (°)	Time period
Tokyo	139.775315	35.619923	1983/05/19-2017/12/31
Honmoku	139.699144	35.435539	1978/08/23-2017/12/31
Dainikaiho	139.744066	35.312913	2004/02/20-2016/12/31
Izuoshima	139.392261	34.809532	1978/08/23-2017/12/31
Osaka	135.42587	34.6551	1977/01/01-2016/12/31
Daiozaki	136.892271	34.275416	1976/11/13-2017/12/31

Hegurajima	136.923521	37.842592	1977/01/01-2016/12/31
Shionomisaki	135.728214	33.418954	1977/01/01-2017/12/31

Supplementary Table S4 | Selected typhoon cases and associated meteorological observation stations

Typhoon number	Period	Observation stations
8506	1985/06/24 06:00-1985/07/07 18:00	Tokyo, Honmoku, Hegurajima, Shionomisaki
9805	1998/09/12 00:00-1998/09/18 06:00	Izuoshima, Daiozaki
0115	2001/09/03 00:00-2001/09/12 18:00	Honmoku
0709	2007/08/27 18:00-2007/09/08 00:00	Tokyo
0918	2009/09/29 06:00-2009/10/11 00:00	Tokyo, Osaka
1115	2011/09/09 12:00-2011/09/24 13:00	Tokyo, Honmoku, Daiozaki, Dainikaiho
1217	2012/09/20 00:00-2012/10/03 00:00	Tokyo, Honmoku, Dainikaiho, Izuoshima, Daiozaki
1721	2017/10/15 06:00-2017/10/23 18:00	Tokyo

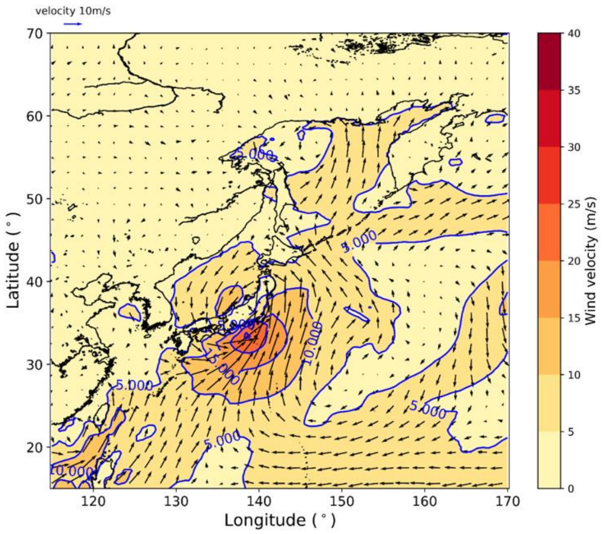

a

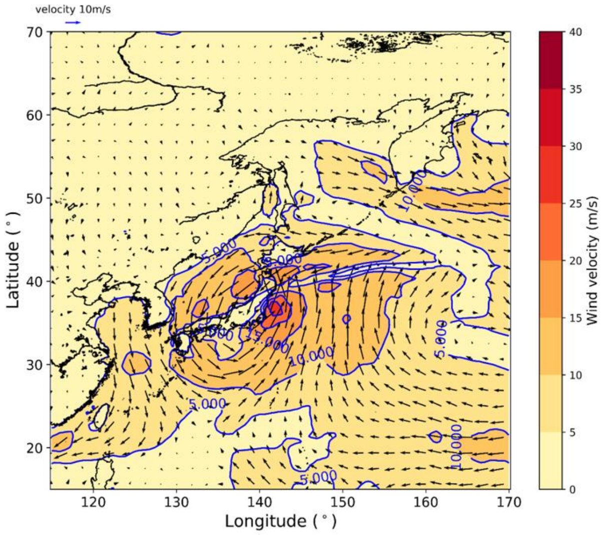

b

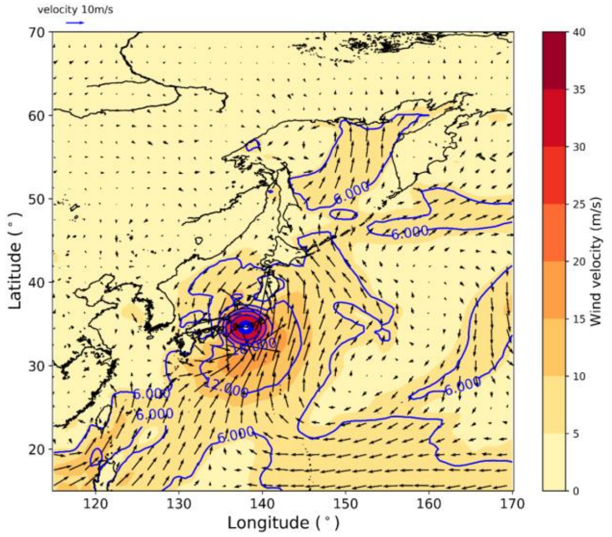

c

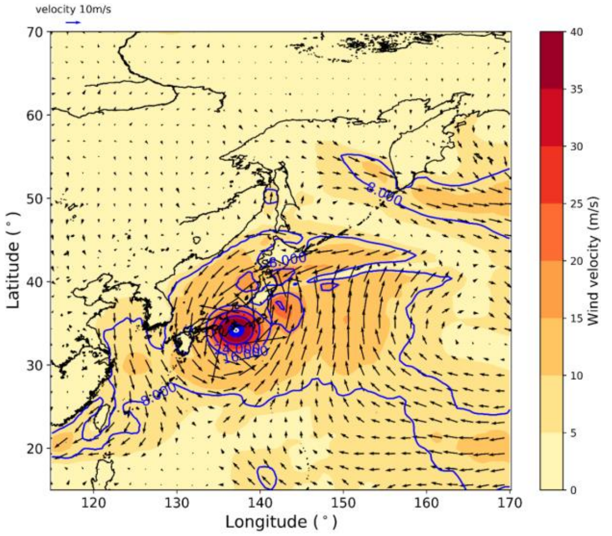

d

Supplementary Figure S1 | Wind velocity distribution using ERA-I and hybrid wind data selecting large values in transition zone at 18:00 on June 30th, 1985, during Typhoon 8506 and at 06:00 on September 21st, 2011, during Typhoon 1115. **a**, ERA-I for Typhoon 8506. **b**, ERA-I for Typhoon 1115. **c**, hybrid wind data for Typhoon 8506. **d**, hybrid wind data for Typhoon 1115.

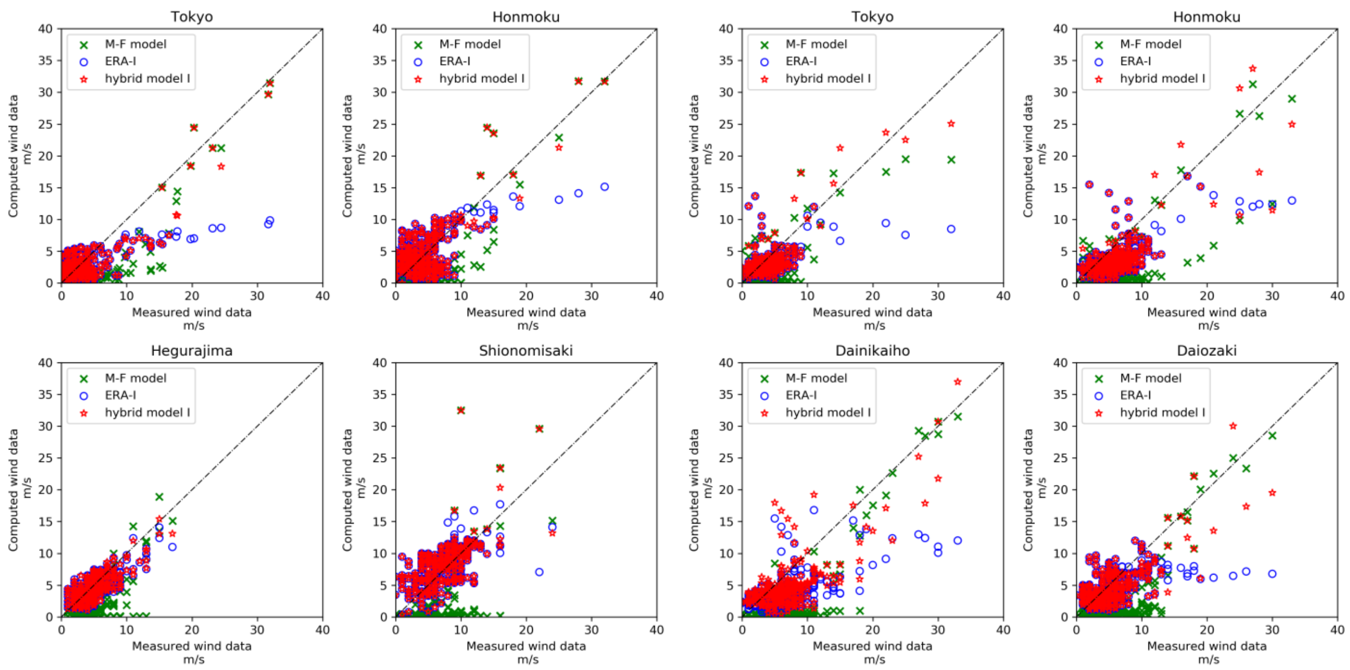

a **b**

Supplementary Figure S2 | Scatter plots of computed wind data (ERA-I, M–F model and hybrid model I) and JODC measured data during two historical typhoons. **a**, Typhoon 8506. **b**, Typhoon 1115. (the legend of Shionomisaki subplot is the same as those of other subplots in **a**)

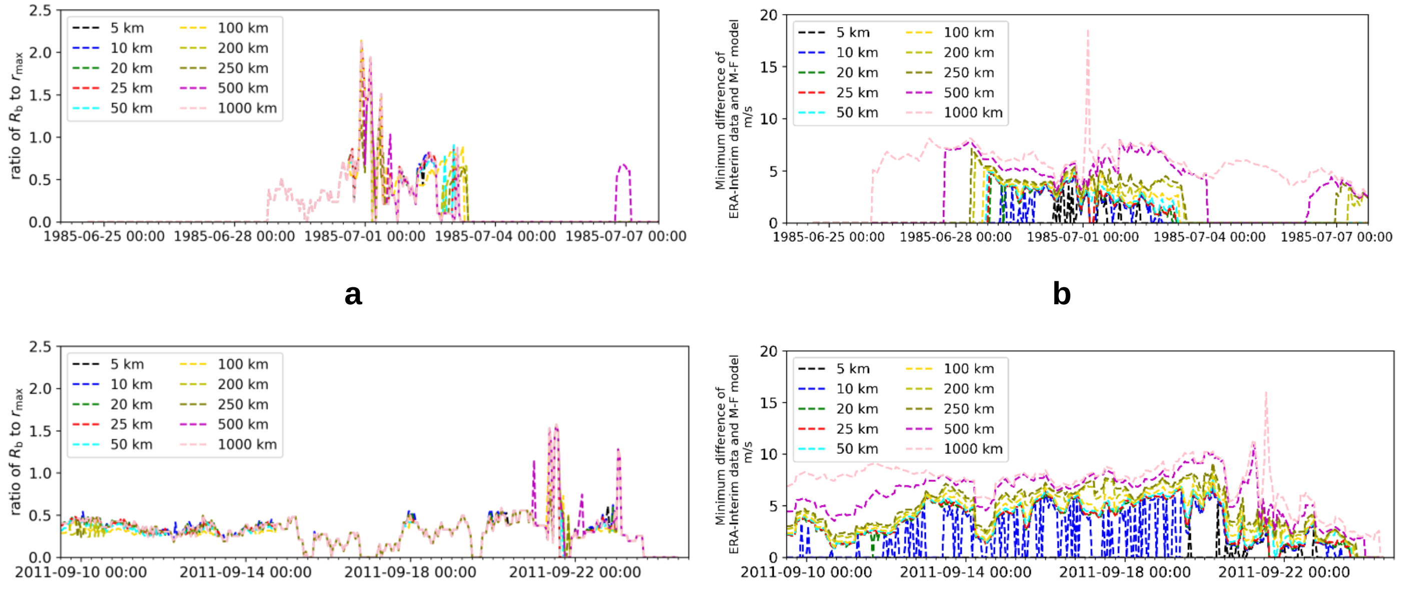

c **d**

Supplementary Figure S3 | Time series ratio of R_b to r_{max} and the minimum difference of ERA-I and M–F model computed using hybrid model II for different bandwidths. **a**, ratio of R_b to r_{max} for Typhoon 8506. **b**, minimum difference of ERA-I and the M–F model for Typhoon 8506. **c**, ratio of R_b to r_{max} for Typhoon 1115. **d**, minimum difference of ERA-I and M–F model for Typhoon 1115.

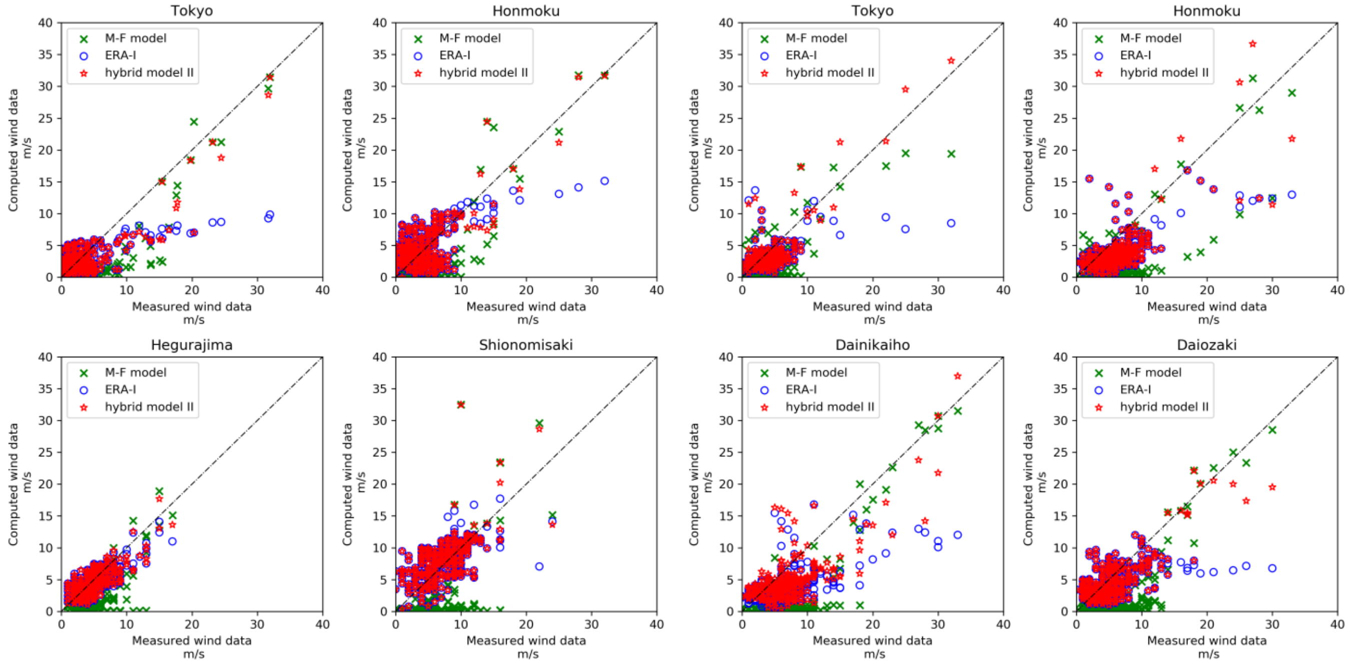

a **b**

Supplementary Figure S4 | Scatter plots of computed wind data (ERA-I, M–F model and hybrid model II) and JODC measured data during two historical typhoons. **a**, Typhoon 8506. **b**, Typhoon 1115. (the legend of Shionomisaki subplot is the same as those of other subplots in **a**)

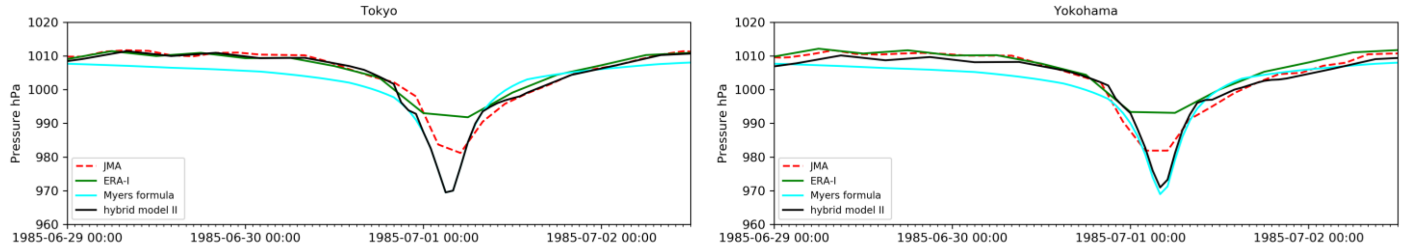

a

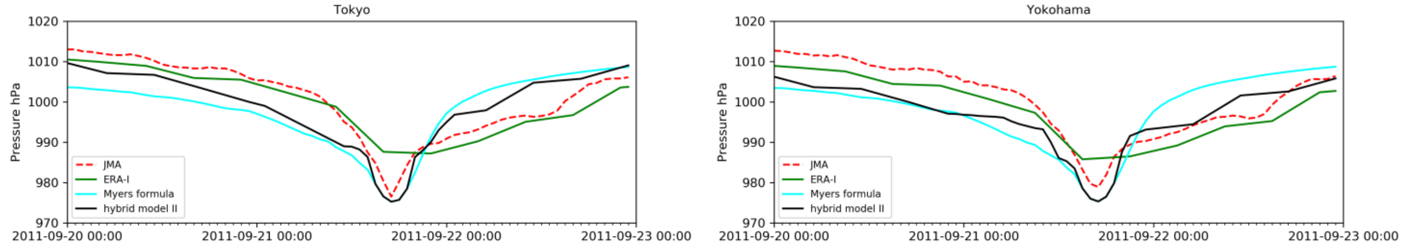

b

Supplementary Figure S5 | Comparison of computed atmospheric pressure (ERA-I, Myers formula and hybrid model II) and Japan Meteorological Agency (JMA) measured pressure data at Station Tokyo and Station Yokohama for two historical typhoons. **a**, Typhoon 8506. **b**, Typhoon 1115. (JMA: Observed atmospheric pressure data from JMA; ERA-I over-predict the atmospheric pressure at 05:00 on July 1st, 1985 for Typhoon 8506 and the atmospheric pressure at 18:00 on September 21st, 2011 for Typhoon 1115)

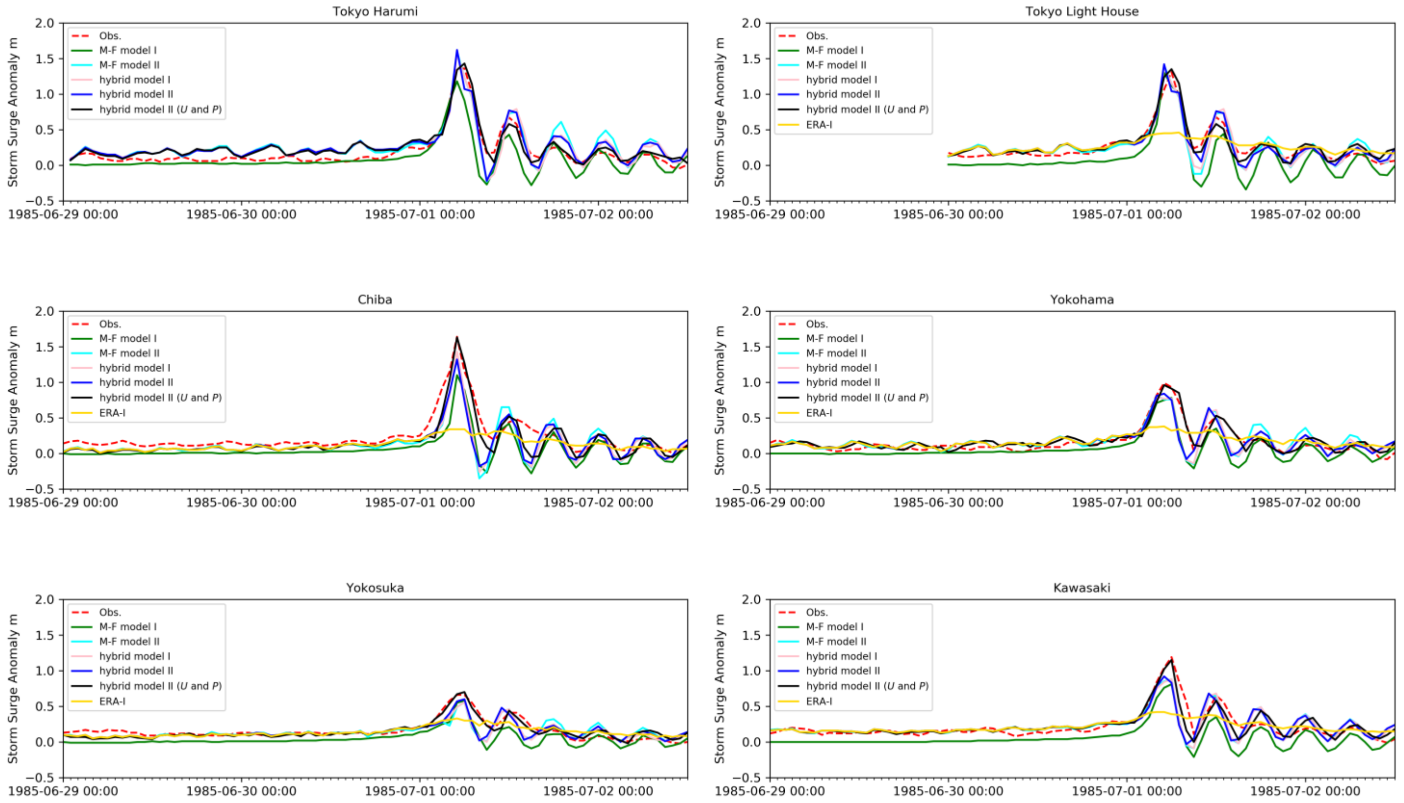

Supplementary Figure S6 | Time series comparison of storm surge simulation results based on different wind and pressure data for Typhoon 8506. (Obs.: Measured storm surge anomaly; M–F model I: storm surge model using wind data computed by the M–F model and pressure data computed by Myers formula; M–F model II: storm surge model using wind data computed by the M–F model and pressure data provided by ERA-I; Hybrid model I: storm surge model using wind speed computed by hybrid model I and pressure data provided by ERA-I; Hybrid model II: storm surge model using wind speed computed by hybrid model II and pressure data provided by ERA-I; Hybrid model II (*U* and *P*): storm surge model using wind speed and pressure data computed by hybrid model II; ERA-I: storm surge model using wind and pressure data provided by ERA-Interim reanalysis datasets.)

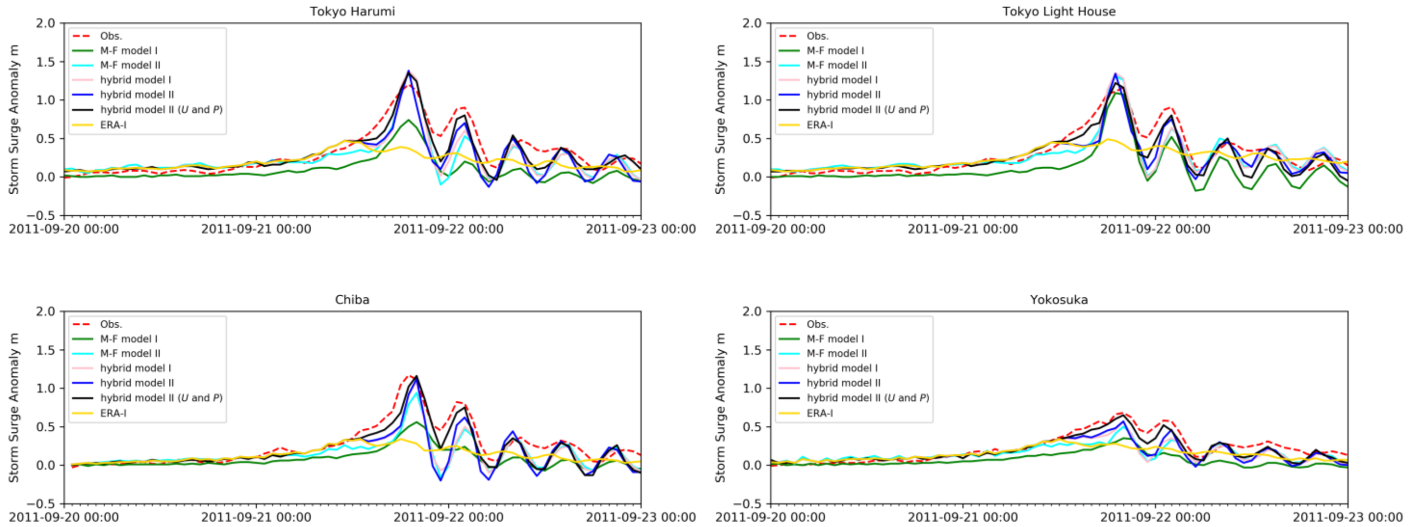

Supplementary Figure S7 | Time series comparison of storm surge simulation results based on different wind and pressure data for Typhoon 1115. (The legends are introduced in Supplementary Fig. S6)
